# Displaced proximal humerus fractures treated with ORIF via the deltoid interfascicular approach vs the deltopectoral approach: A prospective case-control study

**DOI:** 10.1097/MD.0000000000029075

**Published:** 2022-06-10

**Authors:** Bin Liu, Xinguang Wang, Chao Wang, Zhenqing Jiao, Wei Chen

**Affiliations:** a Department of Orthopedic Surgery, The Third Hospital of Hebei Medical University, No. 139 Ziqiang Road, Shijiazhuang, P.R. China; b Hebei Institute of Orthopedic Research, No. 139 Ziqiang Road, Shijiazhuang, P.R. China; c Key Laboratory of Biomechanics of Hebei Province, No. 139 Ziqiang Road, Shijiazhuang, P.R. China; d The Second Hospital of Liaocheng Affiliated to Shandong First Medical University, No. 306 Jiankang Road, Linqing, P.R. China.

**Keywords:** deltoid interfascicular approach, deltopectoral approach, displaced fracture, internal fixation, PHILOS plate, proximal humeral fractures

## Abstract

**Background:**

The purpose of this study was to evaluate the clinical outcomes and complications of displaced proximal humeral fractures treated with proximal humeral internal locking system (PHILOS) plate fixation via a deltoid interfascicular (DI) vs a deltopectoral (DP) approach.

**Methods:**

This prospective case-control study was conducted with patients admitted to our hospital from May 2015 to June 2018 who suffered from unilateral displaced proximal humerus fractures. Patients were treated with PHILOS plate fixation via a DI (DI group) or DP approach (DP group). The clinical outcomes and complication data were collected for comparison between the 2 groups. The patients were followed up at 3, 6, and 12 months; and every 6 months thereafter. The patients’ functional recoveries were evaluated according to the normalized Constant-Murley score, range of motion of the shoulder (flexion, abduction, external/internal rotation) and disabilities of the arm, shoulder and hand score.

**Results:**

A total of 77 patients, followed for an average of 15 ± 2.2months (range, 12–21), were enrolled (36 in DI group and 41 in DP group) for final analysis. No significant differences in age, sex, affected side, fracture type, injury mechanism or time from injury to operation were found between the 2 groups (all *P* > .05). The incision length, intra-operative blood loss, and duration of operation in the DI group were significantly less than those in the DP group, respectively (all *P* < .05). The functional outcomes assessed by the normalized Constant-Murley score and range of motion of flexion and internal rotation in the DI group were superior to those in the DP group at 3 and 6months after the operation (*P* < .05); however, no significant differences were observed at the 12-month and subsequent follow-ups (all *P* > .05). There was no significant difference in the range of shoulder external rotation and abduction during the postoperative follow-ups (*P* > .05). At the last follow-up, the mean disabilities of the arm, shoulder, and hand score was 14.0 (6.6) points in the DI group and 14.4 (6.9) points in the DP group (*P* = .793). Complications occurred in 1 patient in the DI group and 8 patients in the DP group (*P* = .049).

**Conclusion:**

The current study demonstrates that DI approach is a safe and effective alternative for the treatment displaced proximal humerus fractures. The DI approach rather than DP approach was recommended when lateral and posterior exposure of the proximal humerus is required, especially when fixed with PHILOS plate.

## 1 Introduction

Proximal humeral fractures are common shoulder injuries that account for 4% to 5% of all fractures.^[1–3]^ The incidence of these types of fractures is increasing with the ageing society.^[3–6]^ The majority of proximal humeral fractures are non-displaced or slightly displaced and can be successfully treated non-operatively. However, the treatment of displaced or unstable fractures remains a challenge.^[7,8]^ Various methods have been proposed to treat displaced proximal humerus fractures, including conservative treatment, open reduction and internal fixation (ORIF), and shoulder arthroplasty.^[9–11]^ With the use of locking plates, ORIF can achieve excellent fracture reduction, can allow early functional exercise, and is highly recommended by orthopedic surgeons,^[7,12]^ particularly in patients aged younger than 65 years old.^[11]^

The optimal surgical approach for proximal humeral fractures remains controversial. For the past few decades, the 2 most commonly used approaches have been the traditional deltopectoral (DP) approach and the deltoid-splitting (DS) approach. In the DP approach, the lateral and posterior portions of the proximal humerus are difficult to expose, requiring extensive soft tissue dissection and muscle retraction and sometimes release of the deltoid insertion. Poor functional outcomes observed after ORIF performed via the DP approach might be due to the devascularization of fracture fragments during dissection and plating, the disruption of residual blood supply to the head of the humerus or the destruction of the deltoid insertion.^[4,13–15]^ The DS approach has become a new method for exposing the proximal humerus and has also been applied in rotator cuff surgery and intramedullary nailing.^[4,7,13,16–18]^ This approach consists of entering the proximal humerus fracture between the anterior and middle bands of the deltoid. It provides superior exposure of the lateral and posterior portions of the proximal humerus without extensive exposure of the surrounding soft tissue. However, the approach results in a high risk of iatrogenic injury to the axillary nerve, which is considered to be a limitation.^[7,15,19]^

A modified DS approach, named after the deltoid interfascicular (DI) approach, has been proposed for the treatment of displaced proximal humerus fractures.^[16,20]^ This study aims to introduce the DI approach and compare the clinical outcomes and complications of displaced proximal fractures of the humerus treated with ORIF using a proximal humeral internal locking system (PHILOS) plate via the DI approach compared with the DP approach.

## 2 Patients and methods

### 2.1 Study protocol

This prospective case-control study focused on patients with 2-, 3-, or 4-part proximal humeral fractures according to the Neer classification.^[20]^ The inclusion criteria were as follows: aged 18 to 65 years; unilateral closed fracture; acceptance of the suggested ORIF procedure with PHILOS plating via the DP or DI approach; and at least 12 months of follow-up. The exclusion criteria included concomitant fractures of the ipsilateral extremity, pathological fractures, associated neurovascular injuries, severe head injuries, severe cardiopulmonary diseases or neurological diseases, and altered mental status. The patients were divided into the DI group and DP group by a random number table when they were admitted to our department. The patients’ age, sex, affected limb, fracture type, injury mechanism, time from injury to operation and follow-up time were recorded.

### 2.2 Surgical technique

The surgical procedures for all patients were performed by the same senior orthopedic surgeon. The patient was placed in a supine position with a plastic cushion placed under the scapula of the affected extremity. All fractures were treated with ORIF using the PHILOS (Double Medical Technology INC, Xiamen, China).

#### 2.2.1 Deltoid interfascicular approach

**Figure 1:**
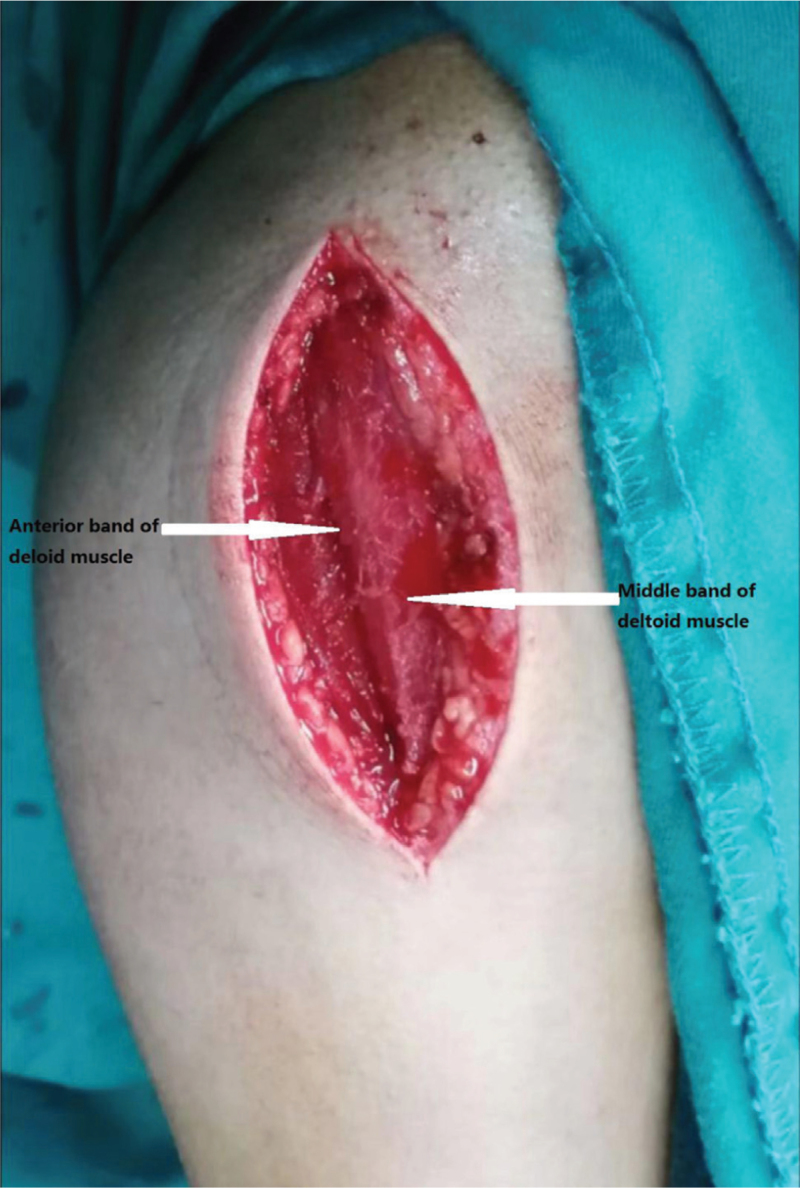
Surgical procedure of deltoid interfascicular approach. The anterior band and middle band of the deltoid muscle is exposed.

**Figure 2:**
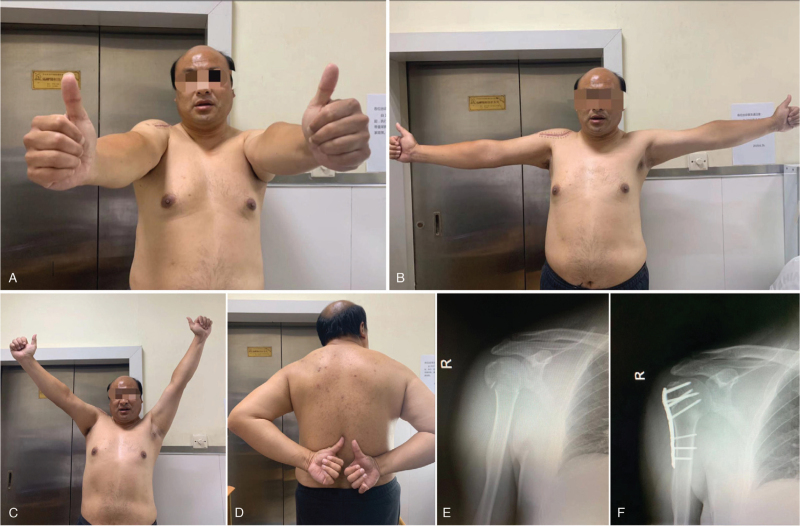
Clinical and functional outcome. (A-D) Function at the 3 mo. (E) Preoperative X-ray. (F) Post-operative X-ray.

A skin incision was initiated in the front of the acromioclavicular joint and extended along the deltoid fibres to the medial margin of the deltoid tuberosity. The fibrous raphe was identified between the anterior and middle bands of the deltoid muscle, and blunt dissection of the deltoid interval was carried out along this raphe (Fig. 1). During the operation, the dissection of soft tissue was minimized, and the anterior circumflex humeral artery was protected. The position of the axillary nerve was identified by anterior and posterior palpation in this interval. Subperiosteal dissection was performed to expose the fracture. Blood clots were removed from the fracture site, and bone fragments were reduced under direct vision. Different reduction techniques were used for different Neer types of fractures. Two-part fractures were reduced by traction of the affected extremity and the tuberosity of the humerus. In 3- and 4-part fractures, a nonabsorbable suture was used to suture the tendon of the rotator cuff at the bone-tendon junction. Reduction of the tuberosity of the humerus was achieved by traction sutures. When a varus fracture occurred in the humeral head, the reduction was accomplished by using a K-wire, and an extractor was used to manipulate the humeral head fragment. Once the fracture was reduced, K-wires were inserted to maintain temporary reduction. The 3–4 hole PHILOS plate was introduced and passed via the interfascicular approach. The plate was placed 2 to 4 mm on the lateral side of the biceps longus tendon, 5 to 8 mm inferior to the greater tuberosity vertex. Five screws were typically inserted into the head of the humerus. In patients with osteoporosis, additional screws were inserted, and 1 to 2 screws of the humerus calcar were inserted. Finally, the 2 ends of the pre-placed sutures were passed through the side holes of the plate and were tied to each other to prevent the displacement of the tuberosity and varus collapse of the humeral head (Fig. 2).

#### 2.2.2 Deltopectoral approach

In the DP approach, a skin incision was initiated at the lateral edge of the coracoid process, running toward the insertion of the deltoid. The cephalic vein was exposed, the deltopectoral groove was blunt dissected, and the long tendon of the biceps was identified. The fracture was reduced, and a 3–4 hole PHILOS plate was placed lateral to the long tendon. The proximal humeral head was fixed with at least 5 mono-cortical locking screws, and the humeral shaft was fixed with 3 to 4 screws.

### 2.3 Postoperative management

Standard anteroposterior (AP) X-rays were taken immediately after the operation, and the neck-shaft angle of the humerus was measured. Sutures were removed 12 days after surgery. All patients underwent the same rehabilitation programme as early as possible postoperatively. They started early controlled passive pendulum movement 24 hours postoperatively and active mobilization exercise 2 to 4 weeks postoperatively, and they started resistance training 6 to 8 weeks postoperatively.

### 2.4 Data collection and efficacy evaluation

The following clinical outcomes were recorded to evaluate between-group differences in perioperative efficacy: incision length, intra-operative blood loss, operative time, postoperative drainage volume, neck-shaft angle, and inpatient stay.

All patients were routinely followed up at 3, 6, and 12 months after operation; and every 6 months thereafter. Functional recoveries evaluation of the patients was undertaken using the normalized Constant-Murley score and the range of shoulder motion (flexion, abduction, external/internal rotation) at 3, 6, and 12 months, postoperatively.^[16,21,22]^ The upper limb function was evaluated according to the disabilities of the arm, shoulder, and hand score at the last follow-up.^[23]^ Infection, cephalic vein injury, subacromial impingement, avascular necrosis, screw penetration, nonunion, and axillary nerve injury complications were recorded.

### 2.5 Statistical analysis

The SPSS 21.0 statistical software package (IBM, Armonk, NY) was used for data analysis. The measurement data in line with a normal distribution were expressed as the mean values ± standard deviations (x ± s), and differences between groups were evaluated by standard t tests. A chi-square test was used to compare the rate and percentage of counting data. *P* values < .05 were considered statistically significant.

### 2.6 Ethical considerations

This study was approved by the Institutional Ethical Board of the 3rd Hospital of Hebei Medical University, and all patients signed informed consent. The study approval number was K2015-00-12.

## 3 Results

Between May 2015 to June 2018, 91 patients were enrolled in this study. Fourteen patients were excluded from the final analysis due to incomplete clinical data (5 patients) or less than 12 months of follow-ups (9 patients). The remaining 77 patients were followed for an average of 15 ± 2.2 months (range, 12-21) (36 in DI group and 41 in DP group). The mean follow-up period was 15.9 ± 2.2 months for DI group and 15.8 ± 2.3 months for DP group. There were no significant between-group differences regrading age, sex, affected limb, fracture type, injury mechanism, time from injury to operation, or follow-up period. All patients achieved fracture union (DI group 12.8 ± 2.3 weeks, DP group 13.6 ± 2.8 weeks, *P* = .194) (Table 1).

**Table 1:** Description and comparison of study population.

	DI group N = 36	DP group N = 41	*t*/*x*^2^ value	*P* value
Age (yrs)	45.1 ± 12.5	45.4 ± 12.6	0.144	.909
Gender (female/male)	23/13	25/16	0.069	.729
Affected limb				
Left	19	24	0.258	.612
Right	17	17		
Neer fracture pattern				
2-part	13	15	0.099	.952
3-part	16	17		
4-part	7	9		
Injury mechanism				
Traffic accident	9	7	1.957	.376
Fall from standing	21	22		
Falling height	16	12		
Time from injury to operation (d)	4.4 ± 1.8	4.9 ± 1.9	1.25	.216
Time of fracture union (wks)	12.8 ± 2.3	13.6 ± 2.8	−1.310	.194
Follow-up (mo)	15.9 ± 2.2	15.8 ± 2.3	0.266	.791

DI = deltoid interfascicular, DP = deltopectoral.

The incision length was 10.8 (2.8) cm in DI group and 12.2 (2.6) cm in DP group, which indicated that DI required a smaller incision length (*P* < .05) than DP. Similarly, DI resulted in less intra-operative blood loss (*P* < .05) when compared to DP (121.9 (48.8) mL vs 150.2 (48.9) mL, respectively). Additionally, shorter operative time was seen in DI group (75.7 (9.1) minute) when compared to DP group (90.9 (10.5) minute) (*P* < .001). The postoperative drainage volume was 46.1 (13.4) mL in DI group and 57.4 (13.2) mL in DP group (*P* < .001). The neck-shaft angle measured on the postoperative AP films and inpatient stay were not significantly different between the groups (*P* > .05) (Table 2).

**Table 2:** Perioperation parameters were compared.

Perioperation parameters	DI group	DP group	T-value	*P* value
Incision length (cm)	10.8 ± 2.8	12.2 ± 2.6	2.39	.019
Blood loss (mL)	121.9 ± 48.8	150.2 ± 48.9	3.43	.013
Duration of operation (min)	75.7 ± 9.1	90.9 ± 10.5	6.72	.000
Postoperation drainage volume (mL)	46.1 ± 13.4	57.4 ± 13.2	3.73	.000
Neck-shaft angle (°)	124.6 ± 7.0	121.9 ± 6.8	1.70	.094
Inpatient stay (d)	10.1 ± 3.3	10.2 ± 2.5	0.20	.842

DI = deltoid interfascicular, DP = deltopectoral.

The normalized Constant-Murley scores were compared between the 2 groups at 3, 6, and 12 months, postoperatively. At 3 months, the score was 59.0 (15.7) in DI group and 50.9 (14.5) in DP group (*P* < .05). At 6 months, the score was 77.4 (13.0) in DI group and 70.0 (15.0) in DP group (*P* < .05). At 12 months, the score was 86.5 (14.9) in DI group and 85.2 (14.8) in DP group (*P* > .05) (Table 3). The DI group had a higher range of flexion and internal rotation of the shoulder than the DP group at the 3 and 6 months follow-ups (*P* < .05), and there was no significant difference from 12 months to the last follow-ups (*P* > .05). There was no significant difference in the range of external rotation and abduction of the shoulder during the postoperative follow-ups (*P* > .05) (Table 4). At the last followup, the mean disabilities of the arm, shoulder, and hand score was 14.0 (6.6) in the DI group and 14.4 (6.9) in the DP group (*P* = .793).

**Table 3:** Normalization and non-normalization Constant-Murley scores were compared at 3, 6, and 12mo of follow-up after the operation.

Constant score	DI group	DP group	T-value	*P* value
Non-normalization				
3 mo	52.6 ± 14.4	45.4 ± 13.6	2.259	.027
6 mo	68.9 ± 12.2	62.6 ± 14.3	2.063	.043
12 mo	76.9 ± 14.2	76.2 ± 14.8	0.242	.810
Normalization				
3 mo	59.1 ± 15.7	50.9 ± 14.5	2.388	.019
6 mo	77.4 ± 13.0	70.0 ± 15.0	2.280	.025
12 mo	86.5 ± 14.9	85.2 ± 14.8	0.359	.721

DI = deltoid interfascicular, DP = deltopectoral.

**Table 4:** Comparison of the range of motion of should at 3, 6, and 12mo after operation.

	DI group	DP group	T-value	*P* value
Flexion				
3 mo	84.5 ± 10.4	75.5 ± 10.5	3.35	.001
6 mo	113.1 ± 10.9	108.0 ± 10.4	2.08	.041
12 mo	132.7 ± 11.8	132.1 ± 11.3	0.24	.813
Abduction				
3 mo	90.9 ± 13.6	87.0 ± 15.6	1.17	.248
6 mo	120.2 ± 15.0	118.4 ± 15.7	0.51	.609
12 mo	132.4 ± 16.0	132.3 ± 16.0	0.02	.984
Internal rotation				
3 mo	57.4 ± 9.0	52.1 ± 9.7	2.47	.016
6 mo	69.6 ± 8.4	65.7 ± 8.1	2.05	.043
12 mo	77.8 ± 6.8	77.1 ± 7.5	0.41	.680
External rotation				
3 mo	19.8 ± 5.8	19.5 ± 5.8	0.24	.809
6 mo	33.6 ± 6.6	33.6 ± 6.4	0.02	.984
12 mo	42.9 ± 6.8	42.6 ± 6.3	0.21	.835

DI = deltoid interfascicular, DP = deltopectoral.

The incidence of complications in the DP group was 19.5%, whereas it was 2.8% in the DI group. There were significant differences between the 2 groups (*P* = .049) (Table 5).

**Table 5:** Complication.

Complication	DI group (n)	Solution	DP group (n)	Solution	*x*^*2*^-value	*P* value
Infection	1	Debridement + antibiotics	0			
Avascular necrosis	0		2	1 patient: shoulder arthroscopy after 12 mo		
Screw perforation	0		1	Removal of perfored screw		
Cephalic vein injury	0		1			
Subacromial impingement	0		4	Plate removal		
Nonunion	0		0			
Auxillary injury	0		0			
Total	1 (2.8%)		8 (19.5%)		3.860	.049

DI = deltoid interfascicular, DP = deltopectoral.

## 4 Discussion

In this study, displaced proximal humeral fractures were treated with ORIF via the DI or DP approach. The fractures healed well, and satisfactory clinical outcomes were achieved in all patients. The DI approach leaded to similarly good functional outcomes in comparison to the DP. However, some differences were found and were discussed as follows.

The DP approach is an anterior surgical approach of the shoulder joint, allowing repair of its anterior, inferior, and superior structures. However, exposure to the posterior aspect of proximal humerus will be difficult.^[4,20]^ Forceful deltoid muscle retraction and extensive soft tissue dissection are needed.^[24,25]^ Conversely, the DI approach is a relatively minimally invasive method of ORIF. The approach accesses the acromioclavicular joint and the lateral and posterior structures of the proximal humerus via avascular raphe at the junction between anterior and middle deltoids. It has less soft tissue dissection and deltoid muscle retraction than DP approach.^[4]^ Given these factors, DI approach is associated with less intraoperative blood loss, operative time and postoperative drainage volume. These findings in our study comparable with those in previous studies.^[8,20,26]^ However, these previous studies did not report on the incision length for the different surgical approaches. In this study, the DI approach achieve excellent exposure of the proximal humerus through a smaller surgical incision. In a comparative cadaveric study, Sirisreetreerux et al also found difference in area of exposure between the DS approach and the DP approach. The area of exposure to the proximal humerus was 1404.39 ± 359.45 mm^2^ in the DS approach and 1325.41 ± 509.12 mm^2^ in the DP approach. The average exposure area in the DS approach was significantly larger than that in the DP approach.^[4]^ In our opinion, the incision length in the DI approach is smaller because of an excellent exposure.

Constant-Murley score was used to evaluate functional recoveries.^[7,27,28]^ In this study, the DI group had better normalized Constant-Murley scores than the DP group at the 3 and 6 months follow-ups. This was consistent with previous reports by Lin et al.^[28]^ Hepp et al^[20]^ performed a study with a similar design to ours. They reported that the mean normalized Constant-Murley score in the DS group was higher than that in the DP group at 3 months. This was consistent with our findings. However, according to Hepp and his colleagues’ report,^[20]^ no significant differences were found in the normalized Constant-Murley scores between both groups (DS group 69.4 vs DP group 71.4) at 6 months, and the DP group showed a higher score than the DS group (81 vs 73.1) at the 12-month follow-up, which was different from our study. In our opinion, the functional outcomes of surgical treatment for proximal humeral fractures are related to soft tissue recovery, with the DP approach having more significant damage to surrounding muscles than the DI approach.

The studies reported by Lin et al^[28]^ and Hepp et al^[20]^ showed that the range of motion of the shoulder joint in the DS approach was superior to that in the DP approach at 3 and 6 months. However, there was no significant difference in the mid-term outcomes between the 2 approaches. This was also observed in our study. At 3 and 6 months, the range of flexion and internal rotation of the shoulder joint in the DI group was significantly better than that in the DP group. However, there was no significant difference between the 2 groups at the 12-month follow-up and thereafter. Klepps et al^[29]^ conducted a cadaveric study on the DP approach and showed that partial anterior deltoid insertion release (more than one-fifth) could compromise the function of the anterior deltoid. Hepp et al^[20]^ also suggested that the DP approach may weaken the strength of the deltoid, especially the anterior fibres of the deltoid, leading to shoulder flexion function impairment.

Exposure to the proximal humerus in DS approach is limited by the axillary nerve, which is located 5 mm below the border of the acromion process.^[4]^ The literature reported incidence of axillary nerve injuries ranging from 0% to 33% with the DS approach.^[15]^ However, Visser et al^[30]^ considered axillary nerve injury to be caused by the fracture itself. Hepp et al,^[20]^ Borer et al^[7]^ and Frank et al^[8]^ reported no axillary nerve injury in their studies with the use of the DS approach for ORIF in proximal humeral fractures. In this study, none of the patients in the DI group were found to have clinical neurological signs of axillary nerve injury. This was consistent with previous reports.^[7,8,19,20]^ In our opinion, the DI is a modification of the DS by extending the skin incision further distally beyond the area of the axillary nerve, which is directly visualized and protected.

In this study, that observed 9 complications in 77 patients during the entire study period. The complication rate in the DI group was significantly lower than that in the DP group. The most common complication was subacromial impingement. The incidence of subacromial impingement in the DS approach is reported to be between 0% and 26%.^[7,19,31,32]^ In our study, subacromial impingement occurred in 4 patients, all in the DP group. A recent meta-analysis of 2 surgical approaches for proximal humeral fractures showed that the incidence of avascular necrosis with the DS approach was lower than that with the DP approach.^[33]^ In the current study, 2 patients were observed to have avascular necrosis in the DP group and none in the DI group, which was comparable to the results of other studies.^[8,20]^ In our opinion, the DI approach accesses the lateral aspect of the proximal humerus directly and provides ease of direct lateral proximal humeral plate placement. In addition, the DI approach exposes the lateral surface and posterolateral aspect of the humeral head but does not intend to expose the medial calcar area, where the humeral circumflex artery network is located. Therefore, risks of subacromial impingement and avascular necrosis with this approach should be much lower than the DP approach.

This study has several limitations. First, the sample size is relatively small. Second, the follow-up period is relatively short. It is important to recognize the long-term clinical outcomes of displaced proximal humerus fractures through the DI approach. Therefore, further studies are necessary to obtain a more precise efficacy by studying in larger sample size and longer follow-up. However, a strength of our study is prospective case-control experimental design, which overcomes the selection bias in retrospective studies.

## 5 Conclusion

This study showed no significant differences in the fractures healed and functional outcomes between the DI approach and the DP approach for the treatment of displaced proximal humerus fractures. However, the DI approach was significantly superior to the DP approach in incision length, intraoperative blood loss, operative time, postoperative drainage volume and complications. Thus, the authors recommend using the DI approach rather than the DP approach when lateral and posterior exposure of the proximal humerus is required, especially for fractures fixation with PHILOS plate. Nevertheless, multicenter controlled ran-domized clinical trials are still required to improve clinical decision making.

## Author contributions

**Data curation:** Bin Liu, Xinguang Wang, Chao Wang, Zhenqing Jiao, Wei Chen.

**Formal analysis:** Bin Liu, Xinguang Wang.

**Investigation:** Bin Liu, Xinguang Wang, Chao Wang, Zhenqing Jiao, Wei Chen.

**Software:** Zhenqing Jiao.

**Validation:** Zhenqing Jiao.

**Writing – original draft:** Bin Liu, Zhenqing Jiao, Wei Chen.

**Writing – review & editing:** Zhenqing Jiao, Wei Chen.
